# Anti-aging potential of extracts from *Sclerocarya birrea* (A. Rich.) Hochst and its chemical profiling by UPLC-Q-TOF-MS

**DOI:** 10.1186/s12906-018-2112-1

**Published:** 2018-02-07

**Authors:** Tinotenda Shoko, Vinesh J. Maharaj, Dashnie Naidoo, Malefa Tselanyane, Rudzani Nthambeleni, Eric Khorombi, Zeno Apostolides

**Affiliations:** 1 Department of Chemistry, University of Pretoria, Private Bag X20, Pretoria, 0028 South Africa; 2 Biosciences, Council for Scientific and Industrial Research, P.O. Box 395, Pretoria, 0001 South Africa; 3 Department of Biochemistry, University of Pretoria, Private Bag X20, Pretoria, 0028 South Africa

**Keywords:** Marula, *Sclerocarya birrea*, Anti-collagenase, Anti-elastase, Chemical profile, Anti-aging

## Abstract

**Background:**

Degradation of components of the extracellular matrix such as elastin and collagen by elastase and collagenase accelerates skin aging. Phytochemicals that inhibit the activity of these enzymes can be developed as anti-aging ingredients. In this study, an investigation of the anti-aging properties of *Sclerocarya birrea* (A. Rich.) Hochst (Marula) extracts was conducted in vitro with the aim of developing chemically characterized anti-aging ingredients.

**Methods:**

Marula stems, leaves and fruits were extracted using methanol:dichloromethane (DCM) (1:1). The stems were later extracted using acetone, ethanol, methanol:DCM (1:1) and sequentially using hexane, DCM, ethyl acetate and methanol. The stem ethanol extract was defatted and concentrated. Elastase and collagenase inhibition activities of these extracts and Marula oil were determined using spectrophotometric methods. The chemical profile of the ethanolic stem extract was developed using Ultra-performance-liquid chromatography quadrupole-time-of-flight mass spectrometry (UPLC-Q-TOF-MS) with MassLynx software. Pure standards were used to confirm the identity of major compounds and were screened for anti-elastase and anti-collagenase activity.

**Results:**

Marula stems extracts were the most active as they exhibited anti-elastase activity comparable to that of elafin (> 88%) and anti-collagenase activity as potent as EDTA (> 76%). The leaf extract had moderate anti-elastase activity (54%) but was inactive agains collagenase. Marula fruits and oil exhibited limited activity in both assays. The ethanolic extract of Marula stems was the most suitable based on its acceptability to the cosmetic industry and its anti-collagenase activity (99%). Defatting and concentration improved its antiaging activity and lowered the colour intensity. Six compounds have been tentatively identified in the chemical profile of the ethanolic extract of Marula stems of which four; quinic acid, catechin, epigallocatechin gallate and epicatechin gallate have been confirmed using pure standards. Epigallocatechin gallate and epicatechin gallate were as potent (*p* < 0.05) as EDTA at 5 μg/ml in the anti-collagenase assay.

**Conclusions:**

The ethanolic extract of Marula stems can be developed into an anti-aging ingredient as it exhibited very good in vitro anti-aging activity and its chemical profile has been developed. Epicatechin gallate and epigallocatechin gallate contribute to the anti-aging activity of Marula stem ethanol extract.

**Electronic supplementary material:**

The online version of this article (10.1186/s12906-018-2112-1) contains supplementary material, which is available to authorized users.

## Background

Degradation of major components of the extracellular matrix such as elastin and collagen by the enzymes elastase and collagenase accelerates skin aging. The protein elastin is responsible for skin elasticity while collagen is responsible for both strength and elasticity of the skin. The loss of skin elasticity and strength associated with breakdown of elastin and collagen leads to wrinkle formation which characterizes skin aging [1]. Inhibitors of the elastase and collagenase enzymes and growth promotors of elastin and collagen have the potential to be developed into anti-aging ingredients. Natural compounds from plants have been shown to inhibit the activity of these enzymes and to promote synthesis of elastin and collagen [2–4].

*Sclerocarya birrea* (A. Rich.) Hochst (Marula) a member of Anacardiaceae is an important tree to several African communities [5]. In most southern African countries, the oil from Marula kernel has been extracted for a variety cosmetic purposes including skin moisturising, mantainance of healthy skin, processing into soap, and as a shampoo for dry, damaged and fragile hair [6, 7]. Marula oil was included in a patented formulation which can be applied topically in the vicinity of a wound or scar for treatment and prevention of formation of scar tissue [8]. In another disclosure, a Marula oil product trademarked as Maruline with enhanced antioxidant properties was developed and patented by Aldivia a French company and Phytotrade Africa [9]. Marula leaves are also reported to have been traditionally used to treat acne and other skin conditions [10]. In addition, the essential oil extracted from Marula peel was reported to have been used in cosmetic applications [11]. A recent clinical study evaluated the irritancy potential, occlusivity properties, moisturizing and hydrating effects of Marula oil [7]. The study revealed that the oil provides moisturising, hydrating and occlusive effects after topical application. Although reports of traditional use of Marula oil and other parts of the plant for cosmetic purposes have been made [10], a detailed scientific analysis of the Marula plant for its anti-aging potential and a chemical analysis of the anti-aging Marula extract for quality control purposes has not yet been reported. Quality control of bioactive plant extracts, fractions and ingredients used in formulations is needed to ensure consistency in herbal extracts used as cosmetic ingredients and medicines [12]. Chemical fingerprinting using hyphenated chromatography such as Ultra-Perfomance Liquid Chromatography connected to a high resolution mass spectrometer (UPLC-QTOF-MS) is an internationally acceptable method for controlling the quality of herbal medicines and can be utilized in identification and authentication of herbal products [13]. The chemical profile of Marula bark, roots and leaves and their antioxidant activity was reported by Russo et al. [14]. The chemical analysis using high performance liquid chromatography-tandem mass spectrometry (HPLC-MS/MS) resulted in a total of 36 compounds being identified with flavonoid glycosides abundant in leaf extracts and galloylated tannins prominent in bark and root extracts. In another study reverse-phase HPLC coupled to a Q-TOF-MS was used to evaluate the chemical profile of extracts of Marula bark which led to the identification of a total of 95 compounds classified mainly into organic acids, polyphenols and fatty acid derivatives [15]. Despite the traditional and commercial cosmetic applications of the Marula plant, the inhibition of collagenase and elastase activity of this plant has not yet been reported. In this study, anti-aging properties of the extracts of *S. birrea* based on the in vitro collagenase and elastase inhibition were investigated with the aim of developing a chemically characterized anti-aging ingredient.

## Methods

### Chemicals and reagents

Elastase substrate N-Methoxysuccinyl-Ala-Ala-Pro-Val-*p*-nitroanilide (Sigma, M4765), human leukocyte elastase (Sigma, E8140), 0.1 M of 4-(2-hydroxyethyl)-1-piperazinethanesulfonic acid (HEPES, pH 7.5) (Sigma, H3375) with 0.5 M sodium chloride (Sigma, S5886), standard inhibitor, elafin (Sigma, E7280), Collagenase substrate N-[3-(2-Furyl)acryloyl]-Leu-Gly-Pro-Ala (FALGPA)(Sigma, F5135, tris(hydroxymethyl)-methyl-2-aminoethane sulfonate (TES) (Sigma, T1375) with calcium chloride dihydrate (Sigma, C3881), EDTA as the inhibitor (Sigma, E5134), Citric acid (Sigma, C0759) were procured from Sigma Aldrich. 9.8% DMSO (Sigma, D8418), 4% (*w*/*v*) Ninhydrin (Sigma, N4876), 0.16% (w/v) Tin (II) chloride (Sigma, 208,256) and 50% 2-propanol (Sigma, I9616), 1% *v*/v 10 mg/ml BSA (Life Technologies, 30,036,578) were prepared in double distilled water. Collagenase type 1 from *Clostridium histolyticum* (Life technologies, 17,100-017) was purchased from Life Technologies. The pure standards epigallocatechin gallate (Sigma, E4143), epicatechin gallate (Sigma, E3893), epigallocatechin gallate (Sigma, 93,894), epicatechin (Sigma, 68,097), catechin (Sigma, 43,412) and gallic acid (Sigma, 91,215) were purchased from Sigma Aldrich. Quinic acid (Supelco, 46,944-U) was purchased from Supelco. Extraction solvents were of analytical grade, ethyl acetate (Merck,1,047,748), acetone (Merck,1,047,256), dichloromethane (DCM) (Merck, 1,047,260), methanol (Merck, 1,046,862), ethanol absolute (Merck, 1,048,022), n-hexane (Merck, K47641967608) were procured from Merck. Methanol ultra LC (Romil, UN1230), acetonitrile ultra LC (Romil, UN1648) were purchased from Microsep. Water with 0.1% formic acid LC-MS Ultra Chromasolv (Sigma,101,719,312) was purchased from Sigma Aldrich and filter paper Whatman number 1 (1001-50) was used.

### Marula stems, leaves and fruits collection and extraction

Marula leaves, soft wood side stems or twigs and fruits were collected from Hluhluwe river in Kwazulu Natal, South Africa as part of a CSIR programme to collect and test plants for their anti-aging potential. The plants were identified by the taxonomist M.S. Mothogoane from the South African National Biodiversity Institute (SANBI, Tshwane) where voucher specimens are deposited (PRE 0864882). The collected plant materials were oven-dried at 30-60 °C. Dried plant materials were ground to a coarse powder using a hammer mill and stored at ambient temperature prior to extraction. Ground plant materials were extracted with methanol:DCM (1:1) for 24 h at room temperature. The solvent was evaporated using a rotary evaporator at 50-60 °C and then further dried in a desiccator for 24 h. The extracts were stored in a cold room.

### Marula stems collection and extraction

Marula soft wood stems were harvested from the University of Pretoria experimental farm with the help of the curator Jason Samuels. The identity of the plant was confirmed by the curator and taxonomist Prof A.E. van Wyk of the herbarium of the University of Pretoria (voucher number PRU 123535).The plant material was diced into small pieces and air dried in the lab. Dried plant material were ground to a coarse powder using a hammer mill and stored in the dark before use. Ground Marula stem samples (100 g) were extracted separately using 1 l of acetone, ethanol and methanol:DCM (1:1) for a total of 3 h with agitation using a magnetic stirrer at room temperature. Filtration was done using a Whatman filter paper number 1 on a buchner funnel and concentrated using a rotor vapor at 50 °C. Sequential extraction was done on another sample (100 g) using 1 l of each of hexane followed by DCM, ethyl acetate and methanol. Extraction was continuous for a total of 3 h with agitation using a magnetic stirrer. The extracts were filtered and concentrated using a rotory evaporator at 50 °C. The seven Marula stem extracts were stored in the dark.

### Marula oil production

Marula oil was obtained from a commercial supplier Phepisa Natural Resources Institute through the CSIR where it was produced through cold press.

### Defatting of Marula stem extract

In order to remove the intense dark brown colour, 100 mg of the Marula stem ethanol extract was defatted by partitioning between 50 ml of water: ethanol (20%:80%) and hexane 50 ml. After separation of the two layers, the water/ethanol was evaporated to dryness using a rotory evaporator and screened for its anti-aging properties.

### Concentration of actives of Marula stem extract

In order to improve the bio-activity of the Marula ethanol stem extract, a method used by Row and Jin [16] to extract catechins from tea was modified. One (1) gram of Marula stem ethanol extract was dissolved in distilled water (100 ml) at 80 °C by shaking in a sonicator for 10 min (Eumax model: UD300 SH-10 L). The solution was extracted with ethyl acetate (3 × 100 ml). The three organic extracts were combined and evaporated to dryness using a rotory evaporator and screened for anti-aging activity.

### Defatting and concentration of actives of Marula stem extract

In order to remove the intense dark brown colour and to also improve the bio-activity of the Marula stem ethanol extract, the method used by Row and Jin [16] to extract catechins from tea was modified. One (1) gram of Marula stem ethanol extract was dissolved in distilled water (100 ml) at 80 °C by shaking in a sonicator for 10 min. The solution was defatted with hexane (3 × 100 ml) then extracted with ethyl acetate (3 × 100 ml). The combined ethyl acetate was evaporated to dryness using a rotory evaporator and screened for anti-aging activity.

### Determination of anti-elastase activity

The method according to Kraunsoe et al. [17] with a few modifications was used to determine anti-elastase inhibition activity. In 96 well plates, 25 μl of HEPES buffer, 25 μl of test samples (1.4 mg/ml) and 25 μl of elastase (1 μg/ml) (0.00125 enzyme units) were added. The blank contained 75 μl HEPES buffer and the control contained 25 μl elastase and 50 μl HEPES buffer. The positive controls contained 25 μl elastase, 25 μl HEPES buffer and 25 μl elafin (10 μg/ml) or 25 μl N-Methoxysuccinyl-Ala-Ala-Pro-Chloro (10 μg/ml). The inhibitors were added in their respective wells. The solvent controls contained 25 μl elastase, 25 μl HEPES buffer and 25 μl of 10% methanol (MeOH). The test samples were added in triplicate and the experiment was done in duplicate. Since extracts contain pigments, it is possible that these may react with the reagents to give false results. Therefore the extract controls were prepared and contained no enzyme nor substrate but 150 μl HEPES buffer and 25 μl of the extract and were treated as the experimental tubes. These were added in triplicate as well. The plates were incubated at room temperature (25 °C) for 20 min. To all the wells 100 μl of N-Methoxysuccinyl-Ala-Ala-Pro-Val-*p*-nitroanilide (1 mM) was added and the plates incubated further for 40 min at 25 °C. The final concentration of the dried extract in the test samples was therefore 200 μg/ml. A similar procedure was followed for final concentrations of 100 μg/ml (starting concentration = 0.7 mg/ml), 25 μg/ml (starting concentration 0.175 mg/ml), 20 μg/ml (starting concentration = 0.14 mg/ml), 10 μg/ml (starting concentration = 0.07 mg/ml), 5 μg/ml (starting concentration = 0.035 mg/ml).

For analysis of the results, all the extract controls were subtracted from their experimentals. The control wells (enzyme and substrate) turned yellow while the blank and the inhibitors showed no colour change. Therefore the extracts that showed inhibition on elastase were recognized by low yellow colour intensity or no colour change, which in turn determined the extent of inhibition. The absorbance was read at 405 nm on a Tecan-Infinite 500 spectrophotometer.

### Determination of anti-collagenase activity

Anti-collagenase inhibition was determined according to Moore and Stein’s method [18] and modifications by Mandl et al. [19] being incorporated. In 2 ml tubes, 25 μl of collagenase (1 mg/ml) (5.5 enzyme units), 25 μl TES buffer (50 mM) with 0.36 mM calcium chloride, pH 7.4 and 25 μl of test samples (1.4 mg/ml) were added. The blank contained 75 μl TES buffer and the control contained 25 μl collagenase and 50 μl TES buffer. The positive control contained 25 μl collagenase, 25 μl TES buffer and 25 μl ethylenediaminetetraacetic acid (EDTA)(2 mg/ml), the solvent control contained 25 μl collagenase, 25 μl TES buffer and 25 μl of either 10% methanol (MeOH), 10% dimethylsulfoxide (DMSO) or 30% DMSO. The tubes were incubated in a water bath at 37 °C for 20 min. To all the tubes 100 μl of FALGPA (1 mM) was added and the tubes were incubated further for 60 min at 37 °C. The final concentration of the test samples was therefore 200 μg/ml. A similar procedure was followed for final concentrations of 100 μg/ml (starting concentration = 0.7 mg/ml), 25 μg/ml (starting concentration 0.175 mg/ml), 20 μg/ml (starting concentration = 0.14 mg/ml) and 10 μg/ml (starting concentration = 0.07 mg/ml) and 5 μg/ml (starting concentration = 0.035 mg/ml).

Extract controls were prepared and contained no enzyme nor substrate but 150 μl TES buffer and 25 μl of the extract and were treated as the experimental tubes. For the calculation of results, the extract controls were subtracted from the experimentals.

Before use, equal volumes of citrate buffer (200 mM), pH 5.0 and ninhydrin solution were combined, 200 μl of the solution was added to all the tubes and the tubes were placed in a boiling water bath for 5 min. The control tubes (enzyme and substrate) turned blue and there was no colour change in the blank and positive control (EDTA) tubes. Therefore the extracts that showed inhibition on collagenase were recognized by low blue colour intensity or no colour change, which in turn determined the extent of inhibition. The tubes were left to cool down and 200 μl of 50% isopropanol was added to each tube. The contents in the tubes were then transferred to respective wells in 48 well plates (Corning, 3548) and the absorbance was read at 540 nm on a Tecan-Infinite 500 spectrophotometer. Each test sample was done in triplicate [18, 19].

### UPLC-q-TOF-ms

UPLC was performed using a Waters Acquity UPLC system (Waters Corp., MA USA), equipped with a binary solvent delivery system and an autosampler. The dried ethanol extract was reconstituted in acetonitrile with 0.1% formic acid: water with 0.1% formic acid in a (80%: 20%) ratio to a concentration of 1 mg/ml. The extract was centrifuged at 10000 g for 10 min to remove particles. Separation was performed on a Waters BEH C18, (2.1 mm × 100 mm, 1.7 μm column). The mobile phase consisted of solvent A: 0.1% formic acid in purified water and solvent B: acetonitrile with 0.1% formic acid. The gradient elution was optimized as follows: 5% B (0-0.1 min), 5-95% B (0.1-15 min), 95% B (15-16.50 min), 95-5% B (16.50-17.50 min), 5% B (17.50-20 min). The flow rate was 0.400 ml/min and the injection volume was 5 μl. The column temperature was 40 °C. The extracts and standards were run using this method.

### MS conditions

A Waters Synapt G2 high definition QTOF mass spectrometer equipped with an ESI source was used to acquire negative and positive ion data. The system was driven by MassLynx V 4.1 software (Waters Inc., Milford, Massachusetts, USA) for data acquisition. MS calibration was performed by direct infusion of 5 mM sodium formate solution at a flow rate of 10 μl/min and using Intellistart functionality over the mass range of 50 - 1200 Da. The MS source parameters were set as follows for both the positive and negative mode: Source temperature 110 °C, sampling cone 25 V, extraction cone 4.0 V, desolvation temperature 300 °C, cone gas flow 10 l/h, desolvation gas flow (500 l/h). The capillary was 2.8 kV in the positive and 2.6 kV in the negative modes.

### Acquisition

Throughout all acquisitions, a 2 ng/μl solution of leucine encephalin was used as the lockspray solution that was constantly infused at a rate of 2 μl/min through a separate orthogonal ESI probe so as to compensate for experimental drift in mass accuracy. Trap collision energies were 30 V (high) and (10 V) for the low energy.

### Chemical profiling

Compounds were tentatively identified by generating molecular formulas from MassLynx V 4.1 based on their iFit value, and by comparison of MS/MS fragmentation pattern with that of matching compounds from Metlin, Metfusion, ChemSpider and Massbank libraries. Additionally, acquired accurate masses were compared with those of known compounds in compound databases. Pure standards were used to confirm the presence of selected compounds.

### Statistical procedures

**Fig. 1:**
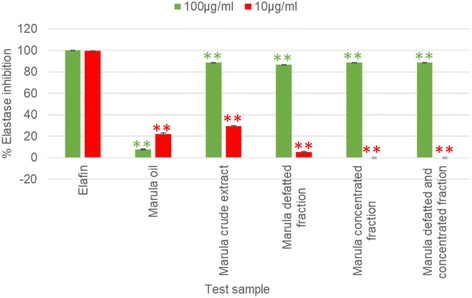
Elastase inhibition of the improved quality samples and Marula oil at 100 and 10 μg/ml (Error bars represent SEM, with *n* = 3).  = *P* < 0.05,  = *P* < 0.01 represent a significant difference with elafin at 10 μg/ml and  = *P* < 0.05,  = *P* < 0.01 represent a significant difference with elafin at 100 μg/ml

**Fig. 2:**
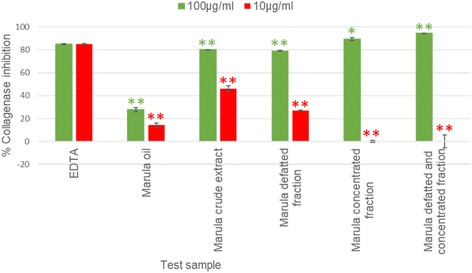
Collagenase inhibition of the fractions with improved quality and Marula oil tested at 100 and 10 μg/ml (Error bars represent SEM, with *n* = 3).  = *P* < 0.05,  = *P* < 0.01 represent a significant difference with EDTA at 10 μg/ml and  = *P* < 0.05,  = *P* < 0.01 represent a significant difference with EDTA at 100 μg/ml

All enzyme assays were done in triplicate. MS Excel was used to analyse the enzyme assay results which were presented as percentage inhibition. The results in Tables 1 and 2 are given as mean ± standard deviation (SD), while the results in Figs. 1, 2 and 4 are shown as mean ± standard error of the mean (SEM). The Student’s t-test function in Excel was used to compare the significance of the differences between the activity of the positive controls and the test samples.

**Table 1:** Comparison of inhibition of the elastase and collagenase for extracts of different parts of *S. birrea*

Plant part	Elastase assay (% Inhibition ±SD)	Collagenase assay (% Inhibition ±SD)
Stems	88.07 ± 2.26	76.92 ± 8.92
Leaves	53.60 ± 1.06	21.23 ± 7.74
Fruits	26.79 ± 6.61	25.89 ± 8.69
	Controls	
10% MeOH	1.45 ± 1.50	1.32 ± 0.73
EDTA		79.88 ± 9.10
Elafin	93.09 ± 4.10

**Table 2:** Extraction yields using different solvents and their inhibition of the elastase and collagenase enzymes

Extraction solvent used for the stems	Extraction yield (calculated from dry plant material) as *w*/w %	Elastase assay (% Inhibition ± SD)	Collagenase assay (% Inhibition ± SD)
Acetone	3.36	** 91.25 ± 0.11	91.17 ± 3.59
Methanol: DCM (1:1)	5.39	** 89.74 ± 0.32	93.75 ± 3.32
Ethanol	3.09	** 93.35 ± 0.89	99.08 ± 3.57
Sequential extraction with hexane	0.38	** 0	** 11.14 ± 2.51
Sequential extraction with DCM	0.22	** 6.69 ± 1.36	** 19.62 ± 2.78
Sequential extraction with ethyl acetate	0.87	** 65.02 ± 1.57	95.56 ± 1.68
Sequential extraction with methanol	8.01	99.19 ± 0.18	94.46 ± 2.31
		Controls	
10% MeOH		3.28 ± 0.34	0
10% DMSO		4.70 ± 0.53	1.58 ± 0.60
Elafin		99.21 ± 0.05	
EDTA			95.66 ± 2.11

** showing a significant difference of *p* < 0.01 with EDTA

** showing a significant difference of *p* < 0.01 with elafin

## Results

### Comparison of activity of different parts of *S. birrea* plant

The results of screening the extracts of Marula stems, leaves and fruits at 200 μg/ml and the positive controls at 100 μg/ml are shown in Table 1. The extract of Marula stems exhibited good anti-collagenase activity of 76.92% and compared favourably to that of EDTA the positive control which had an inhibition activity of 79.88%. In contrast, the extracts of Marula fruits and leaves exhibited limited collagenase inhibition activities of 25.89% and 21.23%, respectively. Similarly, extracts of the Marula stems had very good elastase inhibition activity of 88.07% equivalent to that of the positive control (elafin) which had 93.09%. In comparison, moderate activity was observed for the leaf extract which had anti-elastase activity of 53.60% and very limited anti-collagenase activity of 26.75% for the extract of the fruits.

### Selection of the most suitable extraction solvent

The extraction yields and the inhibition activity of the extracts from the Marula soft wood stems collected from the University of Pretoria experimental farm against the enzymes at test concentration of 200 μg/ml and that of the positive controls at 100 μg/ml is shown in Table 2. The acetone, methanol:DCM (1:1) and ethanol extracts; the hexane, DCM and ethyl acetate sequential extracts exhibited statistically lower (*p* < 0.01) elastase inhibition activities than elafin. In contrast, the methanol sequential extract was as potent as (*p* > 0.05) elafin. Similarly, in the collagenase assay, the hexane and DCM sequential extracts showed significantly (*p* < 0.01) lower activities in comparison to EDTA. On the other hand, the ethyl acetate and methanol sequential extracts; the acetone, methanol:DCM (1:1) and ethanol extracts showed similar (*p* > 0.05) inhibition to EDTA.

### Improving the quality of the ethanol extract as a cosmetic ingredient

The results shown in Fig. 1 revealed that at test concentration 10 μg/ml, the anti-elastase activities of Marula oil, Marula crude extract, Marula defatted fraction, Marula concentrated fraction and the Marula defatted and concentrated fraction were significantly (*p* < 0.01) lower than that of the positive control elafin. A similar trend was observed at 100 μg/ml. Although high elastase activities were exhibited by the Marula crude extract, Marula defatted extract, Marula concentrated fraction and the Marula defatted and concentrated fraction, statistically, these extracts exhibited significantly (p < 0.01) lower potency than the positive control elafin.

At test concentration 10 μg/ml, the collagenase inhibition activities of Marula oil, Marula crude extract, Marula defatted fraction, Marula concentrated extract and Marula defatted and concentrated extract were significantly (*p* < 0.01) lower than that of EDTA. At the higher concentration of 100 μg/ml, Marula oil, Marula crude extract and Marula defatted extract exhibited significantly (*p* < 0.01) lower potency than EDTA (84.81%). However, the Marula concentrated fraction was as potent (*p* = 0.04) as EDTA while the Marula defatted and concentrated fraction was significantly (*p* < 0.01) more potent than EDTA. The colour intensity was lower in the defatted, concentrated and the defatted and concentrated fractions in comparison to the crude stem extract.

### Chemical characterization of the ethanol extract

**Fig. 3:**
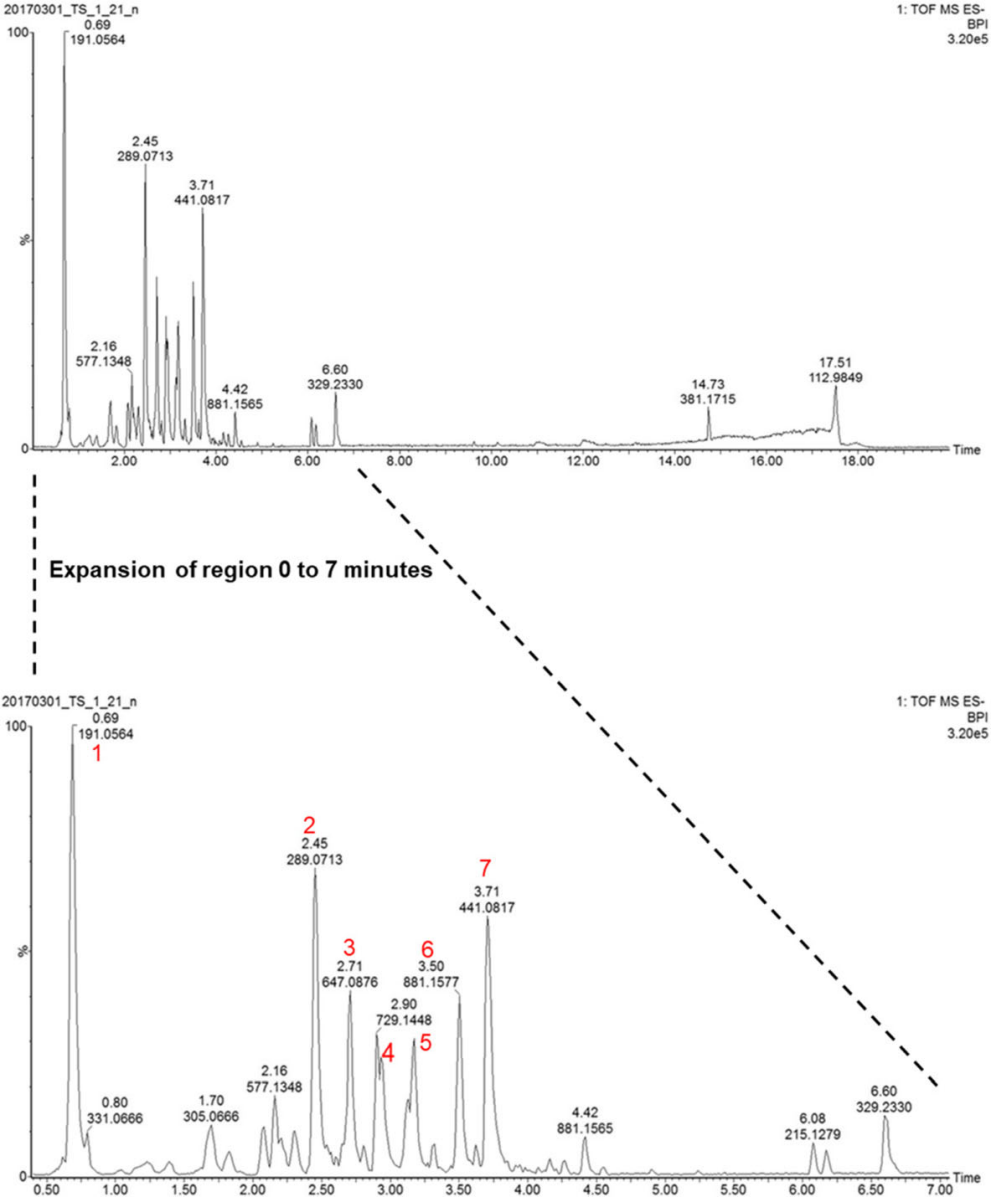
ESI negative mode BPI chromatogram of Marula stem ethanol extract showing the full chromatogram overlaid with an expansion of region 0 to 7 min. A total of six compounds were identified, the presence of four of these; quinic acid (peak 1), catechin (peak 2),epigallocatechin-3-gallate (peak 4), and epicatechin gallate (peak 7) was confirmed using pure standards on UPLC-QTOF-MS. Epicatechin-3-O-gallate-epicatechin (peak 5) and procyanidin B2-3′3 di-O-gallate (peak 6) were tentatively identified and peak 3 was unidentified

The chromatographic profile generated through the analysis of the ethanol extract of Marula stems using the UPLC-Q-TOF-MS operating in the ESI negative mode is shown in Fig. 3 while Table 3 shows the selected compounds that were identified using mass spectrometry.

**Table 3:** Chemical profile of Marula stems extracted with ethanol

Peak #	RT (min.)	Acquired [M-H]^−^ *m/z*	Formula of possible structure	Theoretical [M-H]^−^ *m/z*	Calculated accurate mass (Da)	Possible structure	Mass error (ppm)	MS/MS Data (fragments)		Confirmation with a standard		Ref
										RT (min.)	[M-H]^−^ *m/z*	
1	0.69	191.0564	C_7_H_12_O_6_	191.0561	192.0634	Quinic acid (Organic acid)	−1.57	173.0243	[M-H]^−^ -H_2_O	0.69	191.0555	[25, 30]
								127.0278	[M-H]^−^ - CO-2H_2_O			
								85.0283	[M-H]^−^ - C_3_H_6_O_3_^−^ - H_2_O			
								71.0134	[M-H]^−^ - C_4_H_8_O_3_^−^ - H_2_O			
								59.0145	[M-H]^−^ - C_5_H_8_O_4_^−^			
2	2.45	289.0713	C_15_H_14_O_6_	289.0717	290.0790	Catechin (Flavonoid)	1.38	109.0289	[M-H]^−^ - C_10_H_12_O_3_^−^	2.45	289.0716	[16]
								151.0336	[M-H]^−^ -C_8_H_10_O_2_^−^			
								137.0209	[M-H]^−^ -C_8_H_8_O_3_^−^			
								125.0248	[M-H]-C_9_H_10_O^−^_3_			
								123.0416	[M-H]^−^ -C_8_H_7_O_3_^−^ -H_2_O			
3	2.71	647.0876	C_28_H_24_O_18_	647.0890	647.0963	Unidentified	2.16	495.0740				
								343.0630				
								325.0517				
								169.0133				
								125.0250				
4	2.93	457.0782	C_22_H_18_O_11_	457.0776	458.0849	Epigallocatechin-3-gallate (Flavonoid)	−1.31	305.0670	[M-H]^−^ - C_7_H_4_O_4_^−^	2.93	457.0749	
								289.0657	[M-H]^−^ - C_7_H_4_O_5_^−^			
								169.0142	[M-H]^−^ - C_15_H_12_O_6_^−^			
								125.0243	[M-H]^−^ - C_16_H_13_O_8_^−^			
5	3.17	729.1460	C_37_H_30_O_16_	729.1461	730.1534	Epicatechin-3-O-gallate-epicatechin (Proanthocyanidin	0.14	577.1276	[M-H]^−^ - C_7_H_4_O_4_^−^			[16]
								451.0983	[M-H]^−^ - C_7_H_4_O_4_^−^ -C_8_H_8_O_3_^−^ - H_2_O			
								407.0798	[M-H]^−^ -C_15_H_12_O_6_^−^ -2H_2_O			
								289.0717	[M-H]^−^ - C_7_H_4_O_4_^−^ - C_15_H_12_O_6_^−^			
								169.0147	[M-H]^−^ - C_15_H_12_O_6_^−^ -2H_2_O-C_15_H_13_O^−^_3_			
								125.0240	[M-H]^−^ - C_15_H_12_O_6_^−^ -2H_2_O-C_15_H_13_O_3_^−^ -CO_2_			
6	3.50	881.1577	C_44_H_34_O_20_	881.1571	882.1644	Procyanidin B2-3′3 di-O-gallate (Proanthocyanidin)	−0.68	729.1442	[M-H]^−^ - C_7_H_4_O^−^_4_			[15, 16]
								711.1376	[M-H]^−^ - C_7_H_5_O_5_^−^			
								577.1229	[M-H]^−^ - C_7_H_4_O_4_^−^ - C_7_H_5_O_4_^−^			
								559.1210	[M-H]^−^ - C_7_H_5_O_5_^−^ -C_7_H_5_O_4_^−^			
								407.0760	[M-H]^−^ - C_16_H_14_O_8_^−^ - C_6_H_5_O_3_^−^ - H_2_O			
								289.0716	[M-H]^−^ - C_22_H_17_O_9_^−^ - C_7_H_4_O_5_^−^			
								169.0142	[M-H]^−^ - C_22_H_17_O_9_^−^ - C_6_H_4_O_5_^−^			
								125.0232	[M-H]^−^ - C_22_H_17_O_9_^−^ -C_6_H_4_O_5_^−^ -H_2_O			
7	3.71	441.0817	C_22_H_18_O_10_	441.0827	442.0900	Epicatechin gallate (Flavonoid)	2.27	289.0713	[M-H]^−^ - C_7_H_4_O_4_^−^	3.74	441.0842	[15]
								169.0138	[M-H]^−^ - C_15_H_12_O_5_^−^			
								125.0234	[M-H]^−^ - C_15_H_12_O_6_^−^ -CO_2_			

Using both the ESI negative and positive modes, a total of six compounds were tentatively identified in the extract which included five flavonoids, one organic acid and one unidentified compound. The structure of five of these compounds were confirmed through the use of pure standards which were purchased from commercial suppliers. Additional file 1 shows the ESI positive mode Base Peak Ion (BPI) chromatogram of Marula stems while the full BPI chromatogram of the negative mode and the blank is shown in Additional file 2.

The selection of compounds for identification was based on the peak intensity in chromatography (major peaks) as well mass spectral data which did not show any significant contamination/overlap for compounds which could be co-eluting. The major peak labelled as 1 at *m/z* 191.0564 [M-H]^−^ had fragments which included *m/z* 173 [M-H-18]^−^ due to the loss of a water molecule indicating the presence of an hydroxy group, *m/z* 127 [M-H-28-18-18]^−^ due to subsequent loss of carbon monoxide and two water molecules, indicating the presence of a carboxylic acid group and hydroxy groups respectively (Additional file 3). The identification of quinic acid was confirmed through the comparison of retention times and accurate mass of the standard and peak 1 (Additional file 4).

The chemical profile of Marula stem ethanol extract included several proanthocyanidin dimers, trimers and their flavonoid monomer catechin. Peak 2 with *m/z* 289.0713 [M-H]^−^ fragmented to produce a base peak ion at *m/z* 137 [M-H-152]^−^ due to loss of a pyran ring fragment. The secondary fragments were 151 [M-H-138]^−^ due to loss of a pyran ring fragment, and *m/z* 123 [M-H-152-18]^−^ due to subsequent loss of a pyran ring fragment and a water molecule indicating presence of a hydroxyl group (Additional file 5). Comparison of the accurate mass and retention times of peak 2 and the pure standard confirmed the presence of catechin (Additional file 6). Peak 4 at *m/z* 457.0782 [M-H]^−^ was tentatively identified as epigallocatechin gallate. It was fragmented to produce the base peak ion with *m/z* 169 [M-H-288]^−^ from the loss of a catechin residue. Secondary fragments were obtained at *m/z* 305 [M-H-152]^−^ from the loss of a galloyl residue, *m/z* 289 [M-H-168]^−^ from the loss of a gallic acid unit, *m/z* 125 [M-H-288-44]^−^ due to subsequent loss of a catechin residue and carbon dioxide molecule indicating the presence of a carboxylic acid group (Additional file 7). A comparison of accurate mass measurement and retention time of peak 4 with the pure standard confirmed the presence of epigallocatechin gallate (Additional file 8). Peak 5 at *m/z* 729.1460 [M-H]^−^ was tentatively identified as epicatechin-3-O-gallate-epicatechin. It was fragmented to produce the base peak ion at *m/z* 407 [M-H-152-152-H_2_O]^−^ due to successive loss of a galloyl unit, a pyran ring fragment and a water molecule suggesting the presence hydroxy groups. Secondary peaks were produced at *m/z* 577 [M-H-152]^−^ due to loss of a galloyl residue, *m/z* 289 [M-H-152-288]^−^ from subsequent loss of a galloyl residue and a catechin residue (Additional file 9). A comparison of the theoretical and the experimental accurate masses confirmed the structure. Peak 6 at *m/z* 881.1577 with a retention time 3.50 min was tentatively identified as procyanidin B2-3′3 di-O-gallate. It was fragmented to produce the base peak ion *m/z* 407 [M-H-152-170-152]^−^ due to subsequent loss of a galloyl residue, a gallic acid unit and a pyran ring fragment. Secondary fragments were observed at *m/z* 729 [M-H-152]^−^ due to loss of a galloyl residue, *m/z* 711 [M-H-170]^−^ from the loss of a gallic acid unit, *m/z* 577 [M-H-152-152]^−^ due to loss of two galloyl residue units, *m/z* 559 [M-H-152-170]^−^ due to successive loss of a galloyl residue and a gallic acid molecule. A fragment observed at *m/z* 169 lost carbon dioxide to give another fragment at *m/z* 125 typical of loss of the carbonyl ester (Additional file 10). Accurate mass measurement was used to confirm the molecular formula. Peak 7 observed at *m/z* 441.0817 [M-H]^−^ was fragmented to produce a base peak at *m/z* 169 [M-H-288]^−^ from loss of a catechin residue, secondary peaks were at *m/z* 289 [M-H-168]^−^ due to loss of a galloyl residue, *m/z* 125 [M-H-288- 44]^−^ from subsequent loss of a catechin residue and carbon dioxide (Additional file 11). The compound was tentatively identified as epicatechin gallate. A comparison of accurate mass measurement and retention time of peak 7 and the pure standard confirmed the presence of epicatechin gallate (Additional file 12).

### Collagenase and elastase inhibition activity of pure standards

**Fig. 4:**
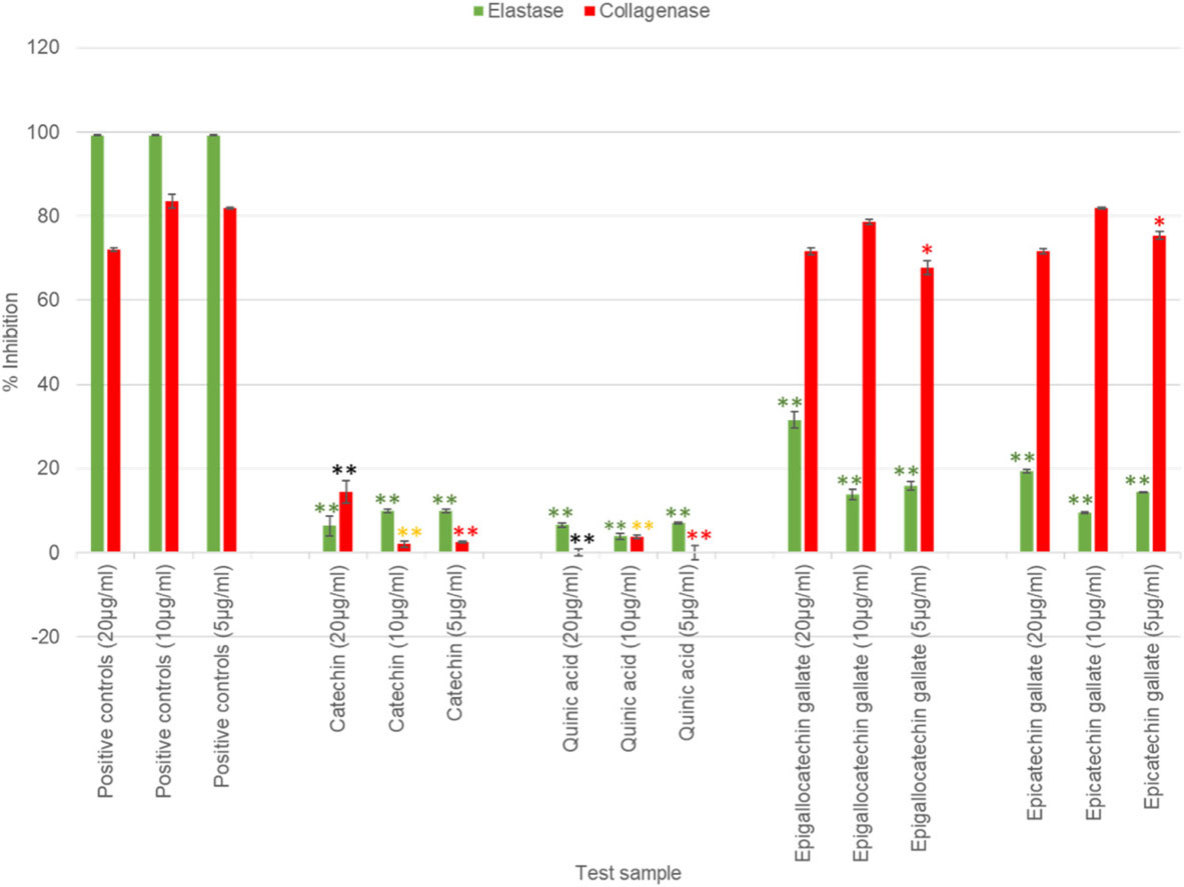
Collagenase and elastase inhibition activities of major compounds identified in Marula stem ethanol extract at 20, 10 and 5 μg/ml. (Error bars represent SEM, with *n* = 3).  = *P* < 0.05,  = *P* < 0.01 represent a significant difference with EDTA at 5 μg/ml,  = *P* < 0.05,  = *P* < 0.01 represent a significant difference with EDTA at 10 μg/ml,  = *P* < 0.05,  = *P* < 0.01 represent a significant difference with EDTA at 20 μg/ml, and  = *P* < 0.05,  = *P* < 0.01 represents a significant difference with elafin

Quinic acid and catechin exhibited significantly (*p* < 0.01) limited activity (< 15%) in both the elastase and collagenase inhibition assays at all test concentrations with respect to the positive controls elafin and EDTA (Fig. 4). In the elastase assay, epigallocatechin gallate and epicatechin gallate exhibited significantly (*p* < 0.01) lower potency than elafin at all test concentrations. At 20 μg/ml and 10 μg/ml epigallocatechin gallate (calculated 44 μM and 22 μM) and epicatechin gallate (calculated 45 μM and 23 μM) showed very good collagenase inhibition activities (> 71%) with potency statistically (*p* > 0.05) equivalent to EDTA. At the lower test concentration of 5 μg/ml (calculated 11 μM), epicatechin gallate and epigallocatechin gallate (> 67%) were in the same potency range (0.01 < *p* < 0.05) as EDTA (81.91%).

### Comparison of chemical profiles of the Marula extract, defatted and concentrated fractions

**Fig. 5:**
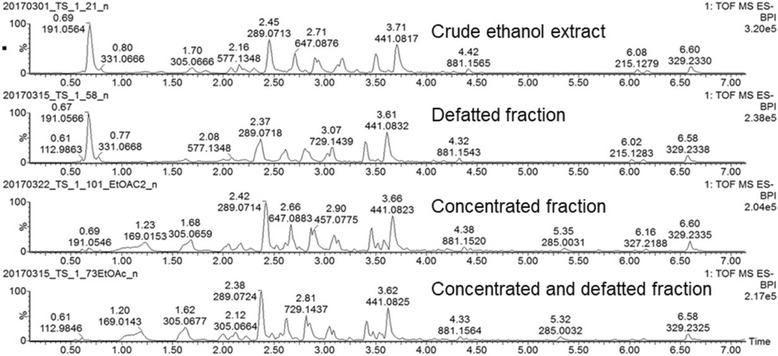
Negative mode BPI chromatograms of Marula stem ethanol extract and its fractions

A comparison of the BPI chromatograms of the Marula extract, defatted and concentrated fractions showed that the major compound identified as quinic acid was removed during the concentration steps of the process (Fig. 5 and Table 4).

**Table 4:** A comparison of major compounds identified in Marula extracts

Compound	RT (minutes)	[M-H]^−^ *m/z*	Crude Ethanol extract	Defatted extract	Concentrated extract	Defatted and Concentrated fraction
Quinic acid	0.69	191.0564	present	present	absent	absent
Catechin	2.45	289.0713	present	present	present	present
Epigallocatechin gallate	2.93	457.0782	present	present	present	present
Epicatechin gallate	3.71	441.0817	present	present	present	present

## Discussion

The extract of the stems showed the most promising inhibition of the two enzymes justifying further research and development. To our knowledge there has been no previous scientific reports on the collagenase and elastase inhibition activity of extracts of the Marula plant.

The development of herbal extracts as ingredients for the cosmetic industry has stringent criteria on the use of approved extraction solvents [20]. As such, a suitable extraction solvent that retained the inhibition activity was investigated. Different solvents were used in order to identify the solvent extracting the most active ingredients. Sequential extraction was done to extract compounds based on polarity. The inhibition activity of the methanol: DCM (1:1) extract of the recollected Marula stems was similar to the earlier collections from Kwazulu Natal. This indicated that the geographical location does not appear to have an effect on the active ingredients. Sequential extraction produced the methanol sequential extract which exhibited potency equivalent to the positive control in the elastase assay. The good elastase inhibition activity of the methanol sequential extract can be attributed to sequential extraction having concentrated the constituents responsible for elastase inhibition thereby producing a potent extract. The general trend revealed by sequential extraction was that the active constituents are concentrated in the highly-polar extracts for collagenase and elastase inhibition. This trend was confirmed in separate extraction in which the highly polar ethanol, acetone and methanol:DCM (1:1) extracts were as potent as EDTA in the collagenase assay. Based on these activities and acceptability to the cosmetic industry, the polar ethanolic extract was selected as the most suitable extract for development as a cosmetic ingredient.

Comparison of the elastase and collagenase inhibition activities of the defatted and the Marula crude stem ethanol extracts to the positive controls, revealed that these two extracts did not show significant activities at all concentrations tested. As such, defatting did not statistically improve the elastase and collagenase inhibition activities of the Marula crude extract. A comparison of the chemical profile of the Marula crude extract with Marula defatted extract revealed that these profiles were similar, which substantiates the lack of improvement in antiaging activity upon defatting the Marula stem ethanol extract.

A significant improvement in collagenase inhibition activity was observed at 100 μg/ml following concentration of the Marula stem ethanol extract. At this concentration, the defatting step subsequently followed by concentration produced an extract exhibiting more potency than that of the original Marula stem ethanol extract, the concentrated extract and the positive control. The high potency observed in this extract is a result of the removal of inactive compounds through both the defatting and concentration steps. The defatted components contribute to the dilution of the active compounds in the separately concentrated extract explaining its lower activity. The separate defatting step and defatting subsequently followed by concentration steps were successful in lowering the colour intensity of the Marula stem extract making it a better cosmetic ingredient.

Marula oil, an ingredient in a variety of cosmetic formulations was also evaluated for its enzyme inhibition for comparative purposes. Our results revealed that Marula oil, although traded as a cosmetic ingredient does not have any anti-collagenase activity contributing to anti-aging claims on products containing the oil. Clinical studies conducted by Komane et al. [7] showed that Marula oil is a non irritant which provides hydrating and moisturising properties to xerotic skin and gives occlusive effects to normal skin. Another study by Mariod et al. [21] showed that Marula kernel oil cake had the highest antioxidant activity in comparison to other parts of Marula. These findings justify the traditional use of Marula oil in cosmetics although it may not be beneficial as an anti-aging ingredient. The extracts of Marula showed good inhibition at the higher concentrations while limited at the low 10 μg/ml. While activity has been seen in the extracts at different concentrations to varying degrees, the chemical compounds in these complex mixtures contribute to efficacy either as single chemical entities or through a combination as synergetic and additive effects [22–24].

The six compounds identified either tentatively or with the use of standards (quinic acid, catechin, epigallocatechin-3-gallate, epicatechin-3-O-gallate-epicatechin, procyanidin B2-3′3 di-O-gallate, epicatechin gallate) in this study have been previously tentatively identified in Marula stem bark in a study by Jimenez et al. [15]. Additionally, in another study by Russo et al. [14] the same compounds from our study were tentatively identified in Marula stem bark with the exception of quinic acid. To date we have no reports for the presence of these compounds in Marula stems. Both quinic acid and catechin were confirmed to be present in the stem extract while previous studies confirmed our findings of the inactivity of quinic acid [25, 26] and catechin [23, 27] in both the elastase and collagenase inhibition tests. A study by Madhan et al. [27] found epigallocatechin gallate to exhibit potent collagenase inhibition activity of 70% at 20 μM which was similar to our results in which epigallocatechin gallate showed 71% collagenase inhibition at 22 μM. A number of studies use epigallocatechin gallate as a positive control in tests for collagenase inhibition and in one of such study it was shown to have an IC_50_ of 12.9 μM [28]. Makimura et al. [23] found epicatechin gallate to be a potent anti-collagenase inhibitor at 113 μM. Our results have shown that epigallocatechin gallate (with > 65% inhibition at 11 μM) and epicatechin gallate (> 75% inhibition at 11 μM) have similar potent anti-collagenase activities and further the compounds are in the same potency range as EDTA a known collagenase inhibitor. The study conducted by Makimura et al. [23] revealed that the steric structure of 3-galloyl radical is important for inhibition of collagenase activity. In our study, epicatechin gallate and epigallocatechin gallate exhibited limited elastase inhibition activity at the three test concentrations. Sartor et al. [22], showed epigallocatechin gallate to be a potent inhibitor of leukocyte elastase with an IC_50_ of 0.4 μM while another study confirmed epigallocatechin gallate to have moderate to weak inhibition of elastase with an IC_50_ of 25.3 μM [29]. The variation in anti-elastase activity observed in our study from that reported in literature is difficult to explain as in our case elafin, the positive control gave the inhibitory activity as expected indicating the validity of the assay. Peak 5 and peak 6 had similar fragmentation patterns to the compounds epicatechin-3-O-gallate-epicatechin and procyanidin B2-3′3 di-O-gallate respectively, which were tentatively identified in Marula stem bark [14, 15].

The major compound corresponding to quinic acid was removed during the concentration steps of the process. As quinic acid was inactive in both the anti-elastase and anti-collagenase assays, the concentration step was successful as it removed this major inactive compound. Epigallocatechin gallate and epicatechin gallate were retained in the concentrated, defatted, and the defatted and concentrated fractions thereby contributing to the anti-aging activity. However, the inactivity of the concentrated and defatted extracts at 10 μg/ml may be attributed to the samples being tested at too low a concentration.

## Conclusions

Extracts of Marula stems exhibited the highest in vitro anti-aging activity in comparison to the extracts of the other parts of Marula. The active constituents were concentrated in the more polar ethyl acetate, ethanol and methanol extracts for both the elastase and collagenase inhibition. Based on these activities and acceptability to the cosmetic industry, the ethanol extract of Marula stems was selected as the most appropriate extract for further research and development although the use of water as an extraction solvent could also be further investigated in the future. The harvesting of stems is often seen as being destructive and non-sustainable. To circumvent this, our study utilized the soft wood side stems or twigs which will not result in destructive harvesting. Sequential extraction successfully extracted compounds based on polarity and produced an extract with good antiaging activity. Defatting and concentration lowered the colour intensity and improved the anti-aging activity of the Marula stem ethanol extract. Marula oil, although traded as a cosmetic ingredient did not have any anti-collagenase or anti-elastase activity contributing to anti-aging claims on products containing the oil. Its application in cosmetic formulations is due to a different mode of action. The chemical profile for Marula stems extracted with ethanol was developed for quality control purposes. Six compounds were tentatively identified from Marula stems extracted with ethanol of which four have been confirmed with pure standards. The presence of quinic acid, catechin, epigallocatechin gallate and epicatechin gallate was confirmed using pure standards. Upon in vitro screening in the collagenase assay, epigallocatechin gallate and epicatechin gallate were as potent as EDTA at 5 μg/ml and were found to contribute to the anti-aging activity of the Marula stem ethanol extract.

## Additional files

Additional file 1: Positive mode Base Peak Ion (BPI) chromatogram of Marula stem extract. ESI positive mode BPI chromatogram of Marula stems extracted with ethanol. (PPTX 179 kb)

Additional file 2: Negative mode BPI chromatogram of Marula stem ethanol extract. Full chromatogram of the ESI negative mode BPI chromatogram of Marula stems extracted with ethanol overlaid with the solvent blank. (PPTX 95 kb)

Additional file 3: MS/MS fragmentation pattern of quinic acid pure standard overlaid with MS/MS fragmentation of peak 1. A comparison of MS/MS fragmentation pattern of quinic acid pure standard and MS/MS fragmentation pattern of peak 1 identified as quinic acid. (PPTX 195 kb)

Additional file 4: Negative mode BPI chromatogram of quinic acid pure standard overlaid with that of Marula stem ethanol extract. A comparison of the retention time of quinic acid pure standard with that of peak 1 identified a quinic acid in Marula stem ethanol extract. (PPTX 85 kb)

Additional file 5: MS/MS fragmentation pattern of catechin pure standard overlaid with MS/MS fragmentation of peak 2. A comparison of the MS/MS fragmentation pattern of catechin pure standard with the MS/MS fragmentation pattern of peak 2 identified as catechin. (PPTX 86 kb)

Additional file 6: Negative mode BPI chromatogram of catechin pure standard overlaid with that of Marula stem ethanol extract. A comparison of the retention time of catechin pure standard with that of peak 2 identified as catechin in Marula stem ethanol extract. (PPTX 86 kb)

Additional file 7: MS/MS fragmentation pattern of epigallocatechin gallate pure standard overlaid with MS/MS fragmentation of peak 4. A comparison of MS/MS fragmentation pattern of epigallocatechin gallate pure standard with that of peak 4 identified as epigallocatechin gallate in Marula stem ethanol extract. (PPTX 140 kb)

Additional file 8: Negative mode BPI chromatogram of epigallocatechin gallate pure standard overlaid with that of Marula stem ethanol extract. A comparison of the retention time of epigallocatechin gallate pure standard with that of peak 4 identified as epigallocatechin gallate in Marula stem ethanol extract. (PPTX 95 kb)

Additional file 9: MS and MS/MS fragmentation pattern of peak 5. An overlay of MS and MS/MS fragmentation pattern of peak 5 tentatively identified as epicatechin-3-O-gallate-epicatechin. (PPTX 82 kb)

Additional file 10: MS and MS MS fragmentation pattern of peak 6. An overlay of MS and MS/MS fragmentation pattern of peak 6 tentatively identified as procyanidin B2-3,3′ di-O-gallate. (PPTX 175 kb)

Additional file 11: MS/MS fragmentation pattern of epicatechin gallate pure standard overlaid with MS/MS fragmentation of peak 7. A comparison of the MS/MS fragmentation pattern of epicatechin gallate pure standard to that of peak 7 identified as epicatechin gallate in Marula stem ethanol extract. (PPTX 84 kb)

Additional file 12: Negative mode BPI chromatogram of epicatechin gallate pure standard overlaid with that of Marula stem ethanol extract. A comparison of the retention time of epicatechin gallate pure standard with that of peak 7 identified as epicatechin gallate in Marula stem ethanol extract. (PPTX 84 kb)

## Abbreviations

DCM: Dichloromethane
DMSO: Dimethyl sulphoxide
EDTA: Ethylenediaminetetraacetic acid
FALGPA: N-[3-(2-Furyl)acroloyl]-Leu-Gly-Pro-Ala
HEPES: (4-(2-hydroxyethyl)-1-piperazineethanesulfonic acid
HPLC-MS/MS: High performance liquid chromatography-tandem mass spectrometry
RP-HPLC-ESI-QTOF/MS^2^: Reverse Phase High performance liquid chromatography-Electrospray ionization quadrupole-time of flight mass spectrometry
RPM: Revolutions per minute
TES: Tris(hydroxymethyl)-methyl-2-aminoethane sulfonate
UPLC-Q-TOF-MS: Ultra-performance liquid chromatography/ quadrupole-time-of-flight mass spectrometry
