# Supplementary material for: Anti-aging potential of extracts from Sclerocarya birrea (A. Rich.) Hochst and its chemical profiling by UPLC-Q-TOF-MS
Source: BMC Complement Altern Med. 2018 Feb 7;18:54. doi: 10.1186/s12906-018-2112-1 (PMC5804067; doi:10.1186/s12906-018-2112-1)
Supplement: Supplementary file 1 — Positive mode Base Peak Ion (BPI) chromatogram of Marula stem extract. ESI positive mode BPI chromatogram of Marula stems extracted with ethanol. (PPTX 179 kb) [file 12906_2018_2112_MOESM1_ESM.pptx]

## Slide 1
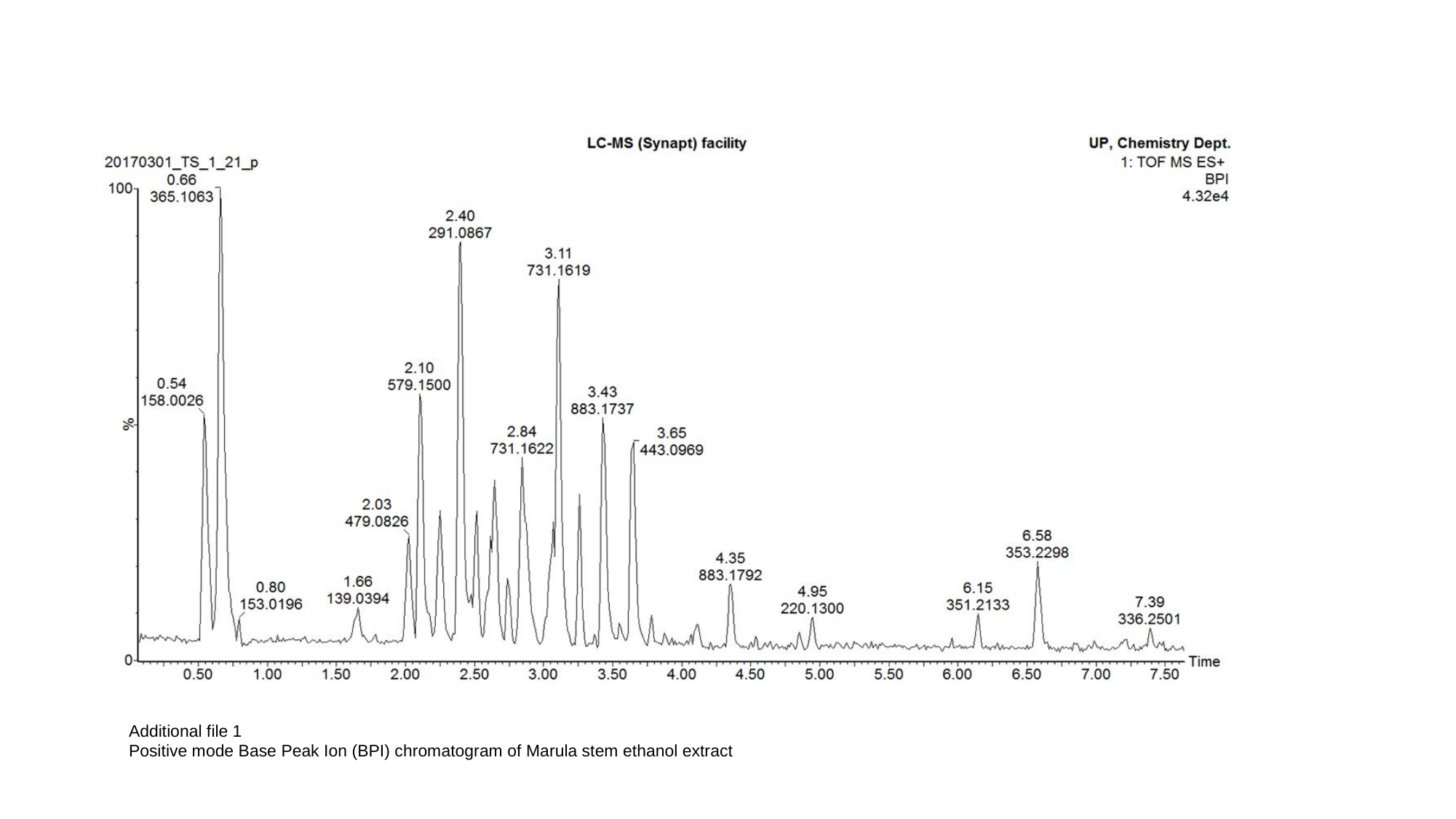

Additional file 1
Positive mode Base Peak Ion (BPI) chromatogram of Marula stem ethanol extract
