# Supplementary material for: Anti-aging potential of extracts from Sclerocarya birrea (A. Rich.) Hochst and its chemical profiling by UPLC-Q-TOF-MS
Source: BMC Complement Altern Med. 2018 Feb 7;18:54. doi: 10.1186/s12906-018-2112-1 (PMC5804067; doi:10.1186/s12906-018-2112-1)
Supplement: Supplementary file 2 — Negative mode BPI chromatogram of Marula stem ethanol extract. Full chromatogram of the ESI negative mode BPI chromatogram of Marula stems extracted with ethanol overlaid with the solvent blank. (PPTX 95 kb) [file 12906_2018_2112_MOESM2_ESM.pptx]

## Slide 1
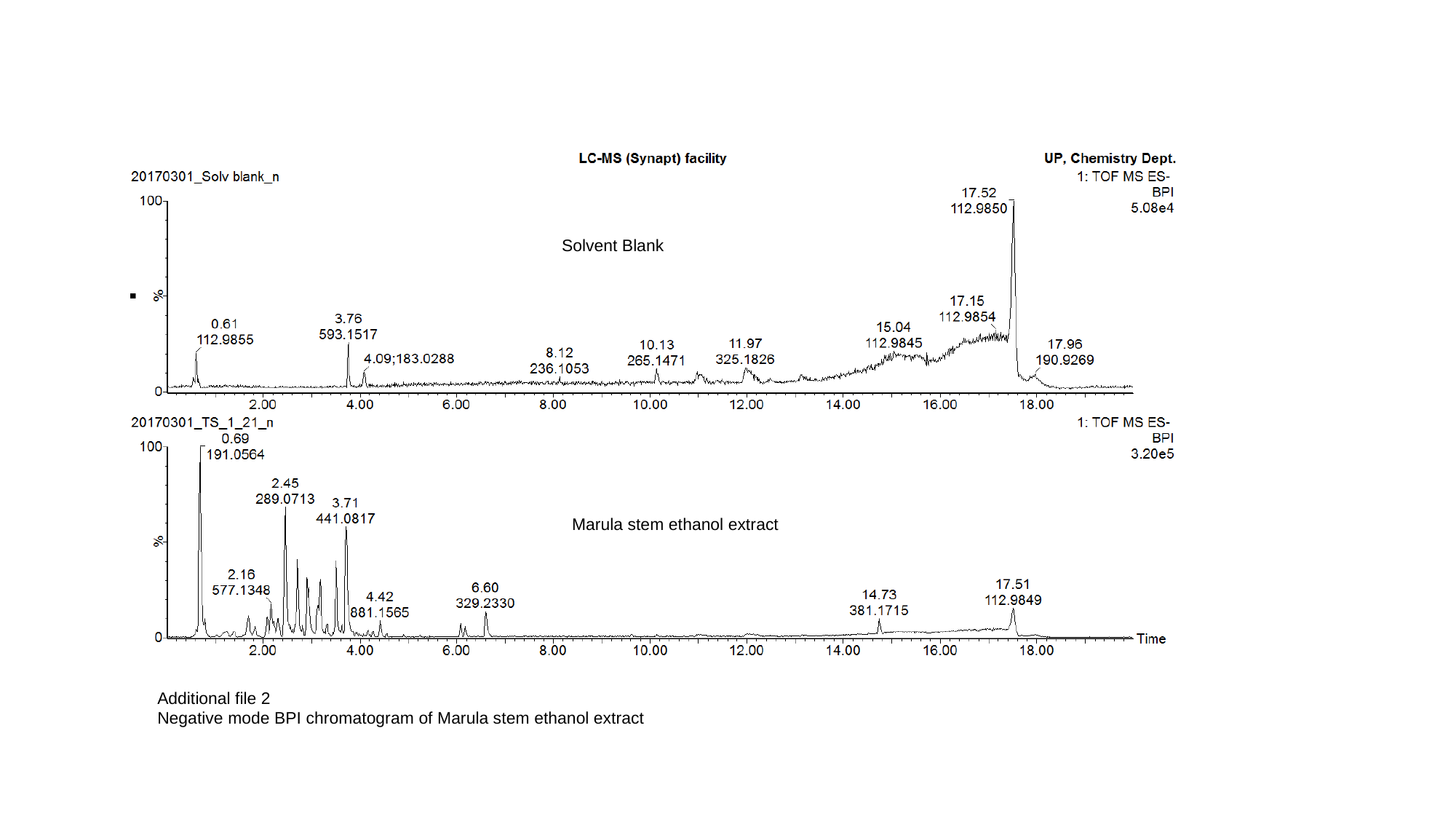

Solvent Blank
Marula stem ethanol extract
Additional file 2
Negative mode BPI chromatogram of Marula stem ethanol extract
