# Supplementary material for: Anti-aging potential of extracts from Sclerocarya birrea (A. Rich.) Hochst and its chemical profiling by UPLC-Q-TOF-MS
Source: BMC Complement Altern Med. 2018 Feb 7;18:54. doi: 10.1186/s12906-018-2112-1 (PMC5804067; doi:10.1186/s12906-018-2112-1)
Supplement: Supplementary file 3 — MS/MS fragmentation pattern of quinic acid pure standard overlaid with MS/MS fragmentation of peak 1. A comparison of MS/MS fragmentation pattern of quinic acid pure standard and MS/MS fragmentation pattern of peak 1 identified as quinic acid. (PPTX 195 kb) [file 12906_2018_2112_MOESM3_ESM.pptx]

## Slide 1
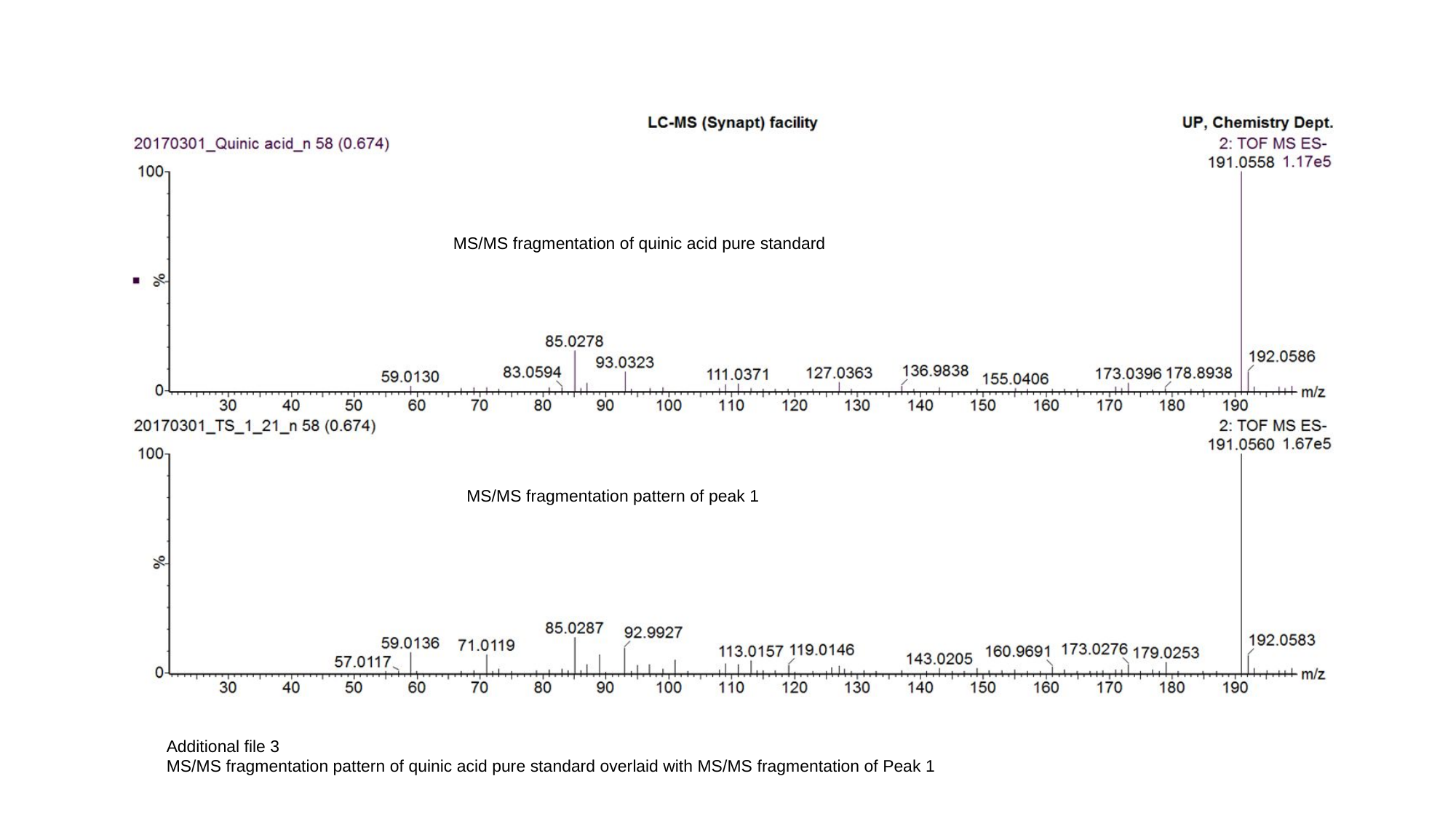

MS/MS fragmentation of quinic acid pure standard
MS/MS fragmentation pattern of peak 1
Additional file 3
MS/MS fragmentation pattern of quinic acid pure standard overlaid with MS/MS fragmentation of Peak 1
