# Supplementary material for: Anti-aging potential of extracts from Sclerocarya birrea (A. Rich.) Hochst and its chemical profiling by UPLC-Q-TOF-MS
Source: BMC Complement Altern Med. 2018 Feb 7;18:54. doi: 10.1186/s12906-018-2112-1 (PMC5804067; doi:10.1186/s12906-018-2112-1)
Supplement: Supplementary file 6 — Negative mode BPI chromatogram of catechin pure standard overlaid with that of Marula stem ethanol extract. A comparison of the retention time of catechin pure standard with that of peak 2 identified as catechin in Marula stem ethanol extract. (PPTX 86 kb) [file 12906_2018_2112_MOESM6_ESM.pptx]

## Slide 1
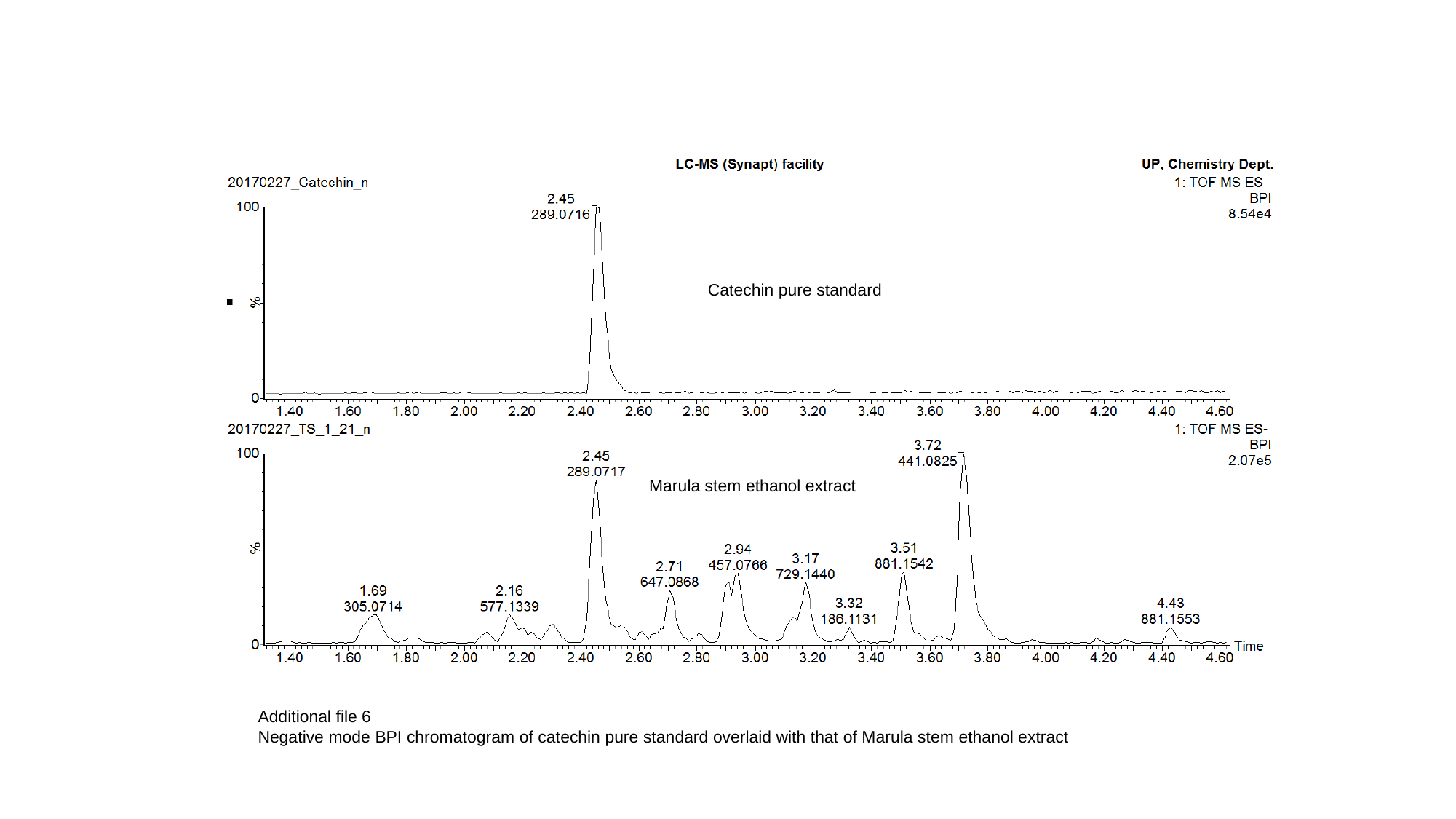

Catechin pure standard
Marula stem ethanol extract
Additional file 6
Negative mode BPI chromatogram of catechin pure standard overlaid with that of Marula stem ethanol extract
