# Supplementary material for: Anti-aging potential of extracts from Sclerocarya birrea (A. Rich.) Hochst and its chemical profiling by UPLC-Q-TOF-MS
Source: BMC Complement Altern Med. 2018 Feb 7;18:54. doi: 10.1186/s12906-018-2112-1 (PMC5804067; doi:10.1186/s12906-018-2112-1)
Supplement: Supplementary file 9 — MS and MS/MS fragmentation pattern of peak 5. An overlay of MS and MS/MS fragmentation pattern of peak 5 tentatively identified as epicatechin-3-O-gallate-epicatechin. (PPTX 82 kb) [file 12906_2018_2112_MOESM9_ESM.pptx]

## Slide 1
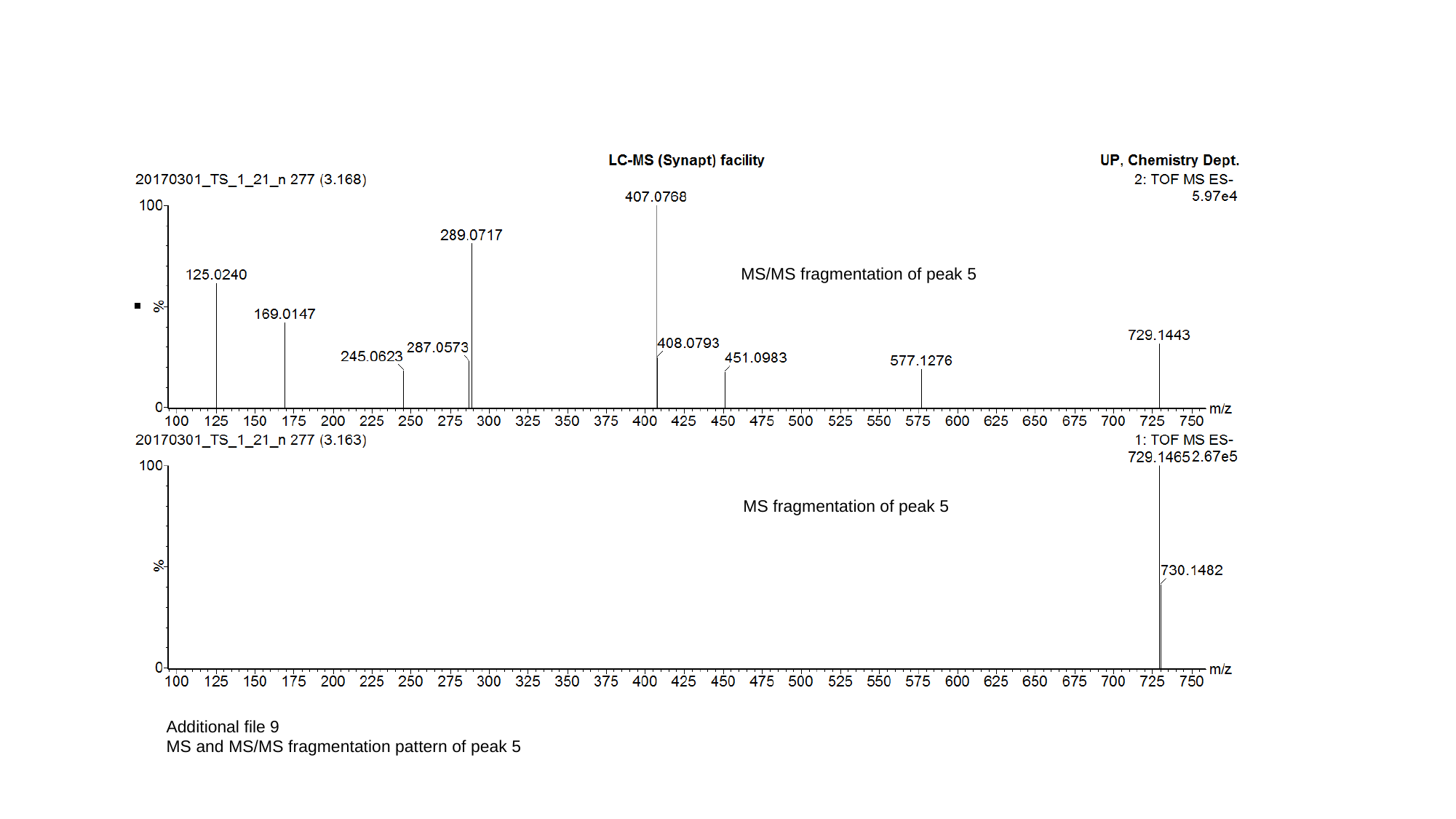

MS/MS fragmentation of peak 5
MS fragmentation of peak 5
Additional file 9
MS and MS/MS fragmentation pattern of peak 5
