# Supplementary material for: Anti-aging potential of extracts from Sclerocarya birrea (A. Rich.) Hochst and its chemical profiling by UPLC-Q-TOF-MS
Source: BMC Complement Altern Med. 2018 Feb 7;18:54. doi: 10.1186/s12906-018-2112-1 (PMC5804067; doi:10.1186/s12906-018-2112-1)
Supplement: Supplementary file 10 — MS and MS MS fragmentation pattern of peak 6. An overlay of MS and MS/MS fragmentation pattern of peak 6 tentatively identified as procyanidin B2-3,3′ di-O-gallate. (PPTX 175 kb) [file 12906_2018_2112_MOESM10_ESM.pptx]

## Slide 1
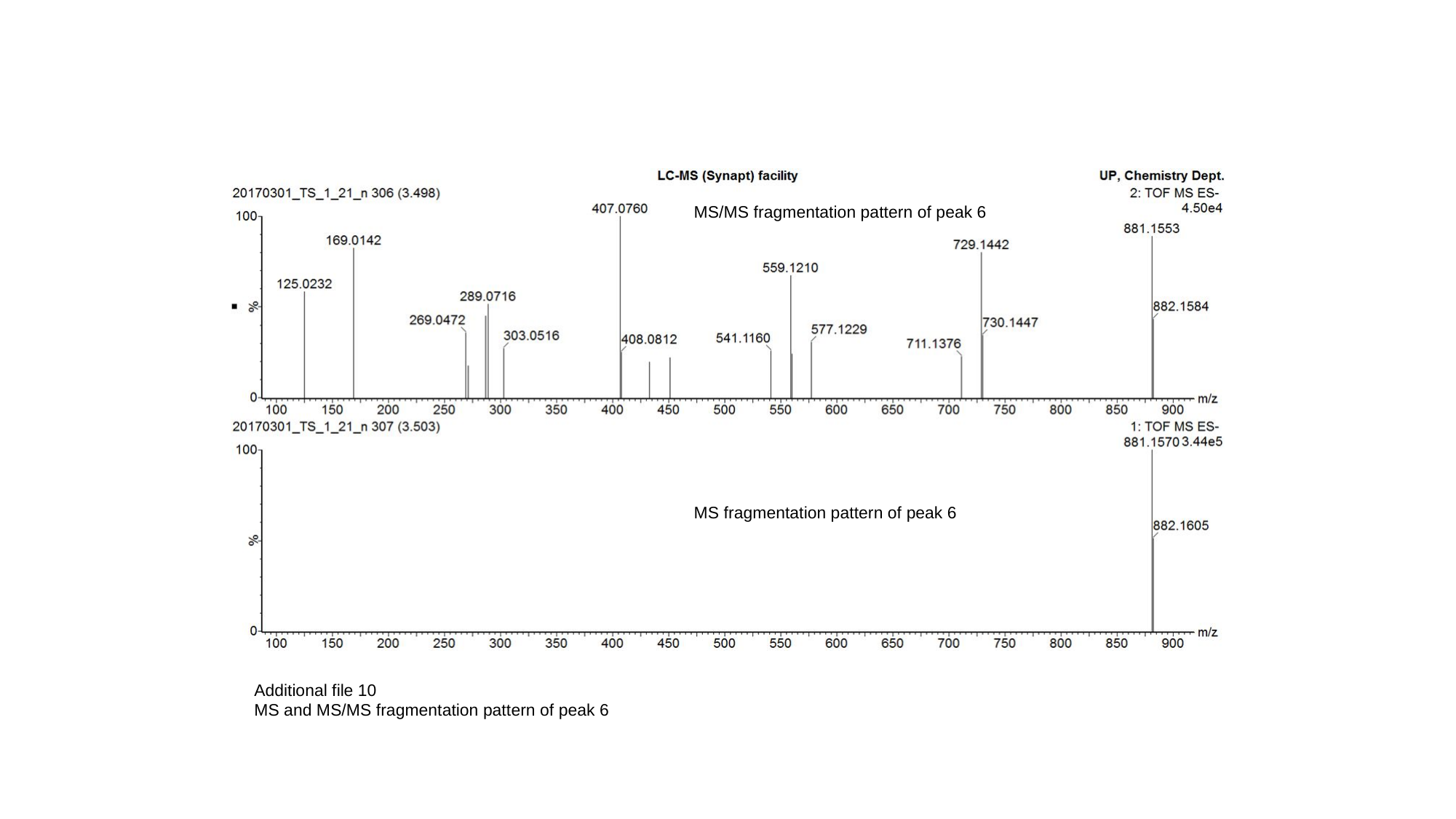

MS/MS fragmentation pattern of peak 6
MS fragmentation pattern of peak 6
Additional file 10
MS and MS/MS fragmentation pattern of peak 6
