# Supplementary material for: Anti-aging potential of extracts from Sclerocarya birrea (A. Rich.) Hochst and its chemical profiling by UPLC-Q-TOF-MS
Source: BMC Complement Altern Med. 2018 Feb 7;18:54. doi: 10.1186/s12906-018-2112-1 (PMC5804067; doi:10.1186/s12906-018-2112-1)
Supplement: Supplementary file 11 — MS/MS fragmentation pattern of epicatechin gallate pure standard overlaid with MS/MS fragmentation of peak 7. A comparison of the MS/MS fragmentation pattern of epicatechin gallate pure standard to that of peak 7 identified as epicatechin gallate in Marula stem ethanol extract. (PPTX 84 kb) [file 12906_2018_2112_MOESM11_ESM.pptx]

## Slide 1
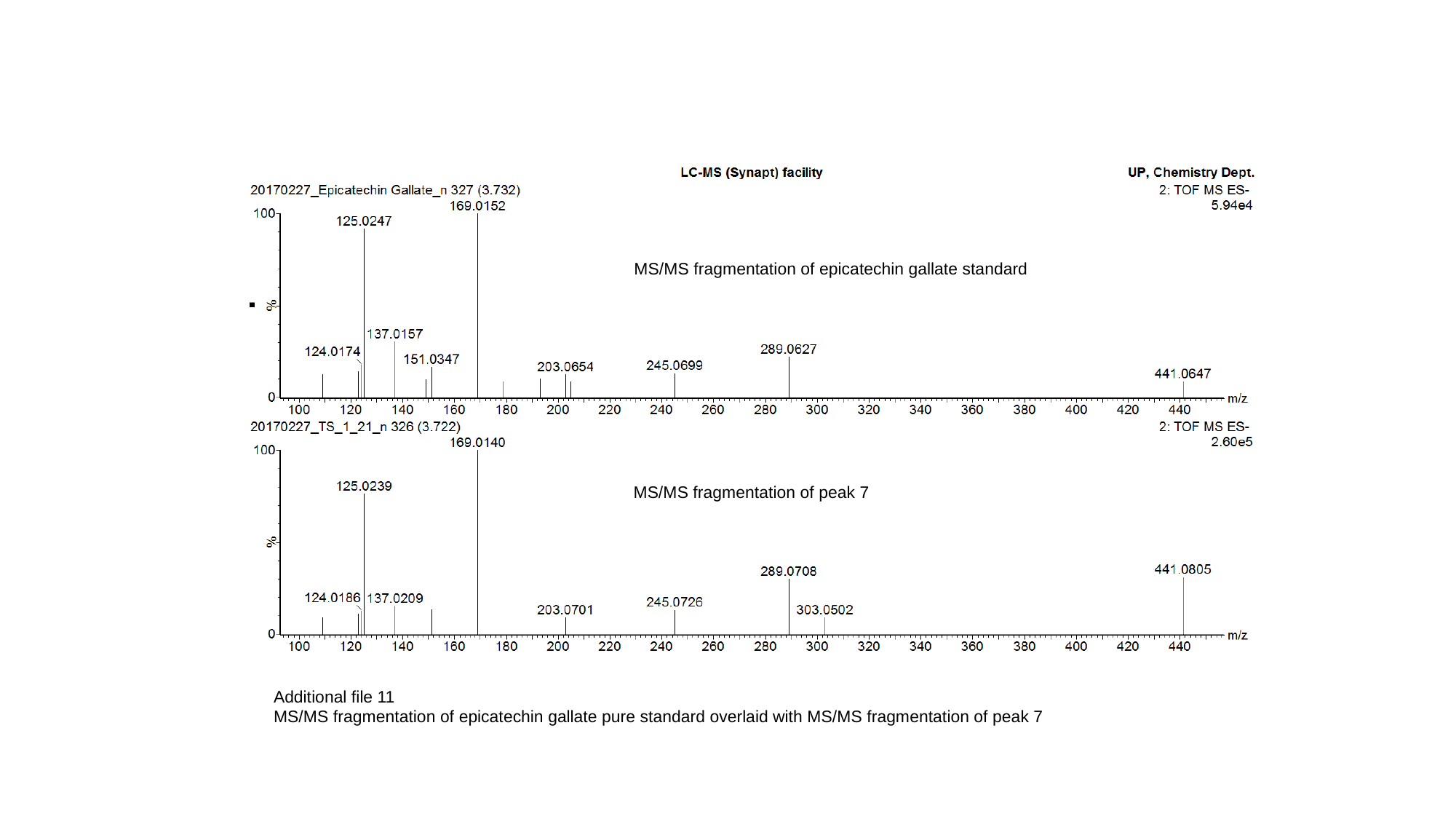

MS/MS fragmentation of epicatechin gallate standard
MS/MS fragmentation of peak 7
Additional file 11
MS/MS fragmentation of epicatechin gallate pure standard overlaid with MS/MS fragmentation of peak 7
